# Development of a Surface Temperature Sensor to Enhance Energy Efficiency Actions in Buildings

**DOI:** 10.3390/s18093046

**Published:** 2018-09-12

**Authors:** Lia Mota, Alexandre Mota, Cláudia Pezzuto, Marcius Carvalho, Marina Lavorato, Lorenzo Coiado, Everton Oliveira

**Affiliations:** Pontifical Catholic University of Campinas, Center of Exact, Environmental and Technological Sciences, CEP 13086-900 Campinas, Brazil; mota.profalexandre@gmail.com (A.M.); claudiapezzuto@puc-campinas.edu.br (C.P.); marcius@puc-campinas.edu.br (M.C.); mlavorato@gmail.com (M.L.); lorenzo.coiado@gmail.com (L.C.); everton_deoliveira20@hotmail.com (E.O.)

**Keywords:** thermal comfort, energy efficiency, surface temperature sensor, temperature monitoring, building energy efficiency rating systems

## Abstract

The air temperature increase in urban centers can lead to problems such as increased energy consumption associated to air conditioning, the intensification of pollution, human discomfort and health problems. In this context, the building envelope plays an important role in urban thermal equilibrium. Energy efficiency rating systems for buildings (LEED—Leadership in Energy and Environmental Design, AQUA—High Environmental Quality, PROCEL Edifica, etc.) stimulate energy efficiency actions in the built environment, considering, for example, the envelope and energy efficiency initiatives in buildings. Research carried out recently has shown that monitoring of buildings can provide important information about building performance, supporting building control strategies and enabling actions aimed at improving energy efficiency and thermal comfort. More specifically, wireless sensors are also being used to monitor buildings. This work proposes and presents the development of a surface temperature sensor that can support actions to enhance energy efficiency in the built environment, meeting the requirements proposed by the energy efficiency rating systems of buildings. This sensor must have characteristics such as low cost, the storage capacity of a large amount of data and the possibility of remote monitoring of the collected temperatures. Computer simulations and validation tests were carried out showing that the proposed sensor allows the remote monitoring (using a wireless transmission system) of the surface temperature in buildings, respecting the requirements of high storage capability and low cost.

## 1. Introduction

The intense urbanization process has led to an increase in air temperature in urban areas, compared to the surrounding rural environment. In this context, Urban Heat Island Effect (UHIE) can be defined as a phenomenon characterized by the elevation of surface temperature in urban areas when compared to the temperature of the surrounding areas. This phenomenon is directly related to some characteristics of the urban environment, such as: large number of buildings, little vegetation presence, heat production by transportation and industry, creation of corridors between buildings (called urban canyons) and the use of materials with high solar absorption [1,2,3,4]. This increase in urban temperature can aggravate energy consumption for cooling purposes, increase peak electricity consumption, intensify pollution problems, cause human discomfort and health problems [1].

In this regard, the materials used in the envelope of buildings play a very important role in urban thermal equilibrium. Its thermal characteristics influence the energy consumption of the buildings and the individual comfort conditions in indoor and in outdoor environments [1]. Thus, the envelope of the building is the key factor that determines and controls the interior conditions. Various components such as walls, surfaces, roofing, thermal insulation, thermal mass, external shading devices, among others, make up this important part of any building [2,5,6,7,8,9,10,11].

Solar radiation has a significant effect on the energy loads of buildings. Recently, many studies [12,13] have been carried out to investigate the effects of thermal insulation of roofs and external walls in building envelopes. Reference [14] reports that one of the possibilities to mitigate the effects of the heat island is the change of the materials used in the construction which, in turn, can lead to direct and indirect beneficial effects for man and the environment as, for example, the reduction of air temperature in urban areas, the reduction of energy consumption by air conditioning systems, among others.

Reference [1] reviews the state of the art on the development and use of “cool materials” (materials of high solar reflectance or high albedo and emissivity) for buildings and conclude that the use of such materials can contribute, significantly, for the mitigation of the urban heat island effects and to the improvement in urban environmental quality.

In this context, energy efficiency rating systems for buildings encourage energy efficiency actions in the built environment, including the monitoring of the envelope of the building, based on quantities such as the ambient temperature and the temperature of the urban surfaces that make up the built environment. Among these energy efficiency rating systems, one can mention Haute Qualité Environnementale des Batiments (HQE, France), BRE Environmental Assessment Method (BREEAM) and ECOHOMES (UK), GREEN STAR rating system (Australia), Comprehensive Assessment System for Building Environmental Efficiency (CASBEE, Japan) and Leadership in Energy and Environmental Design (LEED, USA) [15,16,17,18,19,20,21,22,23].

In Brazil, the concept of sustainable development, thermal comfort and energy efficiency actions are also encouraged by energy efficiency rating systems for buildings, with special emphasis on LEED, High Environmental Quality (AQUA), which is based on the HQE rating system of France [22], and PROCEL Edifica. Thus, both LEED and AQUA rating systems, as well as PROCEL Edifica labeling process, encourage the implementation of initiatives aimed at increasing energy efficiency, which is often linked to the improvement of thermal comfort in existing or new buildings. Consequently, in order to meet the requirements proposed in these rating systems, it is necessary to promote actions aimed at energy efficiency and thermal comfort in the built environment.

Recent studies have shown that building monitoring has provided relevant information on the building’s thermal and energetic performance, offering opportunities for better control strategies in buildings and for actions aimed at energy efficiency and thermal comfort associated with them [24,25]. In this context, wireless sensors are also being used to monitor buildings and their environment [26].

Conventionally, in order to analyze thermal comfort in the built environment, the surface temperature is measured using an equipment composed by a sensor and a data logger for the storage of collected information, as described in Section 2.3. Although these devices are able to efficiently collect temperature measurements, they have as disadvantages their high cost and their limited capacity for information storage, which can often hamper the data collection process. In addition, for this collected information to be analyzed, it must be physically removed (“discharged”) from the storage device, requiring human intervention, so that the collected data can be filtered and analyzed.

In this sense, this work proposes and presents the development of a surface temperature sensor that can subsidize actions to enhance energy efficiency in buildings, meeting the requirements proposed by energy efficiency rating systems of buildings such as LEED, AQUA and PROCEL Edifica.

More specifically, surface temperature monitoring provides subsidies for the analysis of the building envelope and of the thermal comfort. It is important to emphasize that the characteristics of the building envelope and the thermal comfort are directly related to the energy consumption in buildings, since the thermal discomfort promotes a greater use of equipment for controlling the air temperature, like air conditioning systems, that consume a quite significant amount of energy. This means that the monitoring of building surfaces temperatures can help understanding buildings’ behavior and thus can help improving the thermal comfort in the buildings, consequently, reducing the usage of devices for air cooling or heating, promoting a reduction on electrical energy consumption. Hence, surface temperature monitoring (by adequate sensors) favors the requirements that are related to energy consumption reduction in energy efficiency rating systems.

In addition, it is intended to make this sensor accessible (from the point of view of cost and ease of implementation and use) to researchers involved in monitoring buildings and cities. Thus, the sensor proposed in this work was developed based on a concept that is different from the concept observed in the conventionally used sensors (which are basically composed only by a transducer and a data logger). Its design was totally based on components (transducer, microcontroller and wireless transmission system) that allow the sensor to be easily and widely used even by professionals and researchers that are not familiar with wireless monitoring systems. This design concept takes into account the possibility of remote monitoring different surfaces simultaneously, what is not possible using the conventionally employed sensors. In this sense, it is important to emphasize that the proposed sensor has as main advantages, when compared to the ones conventionally used for the same purpose (surface temperature monitoring): low cost, large storage capacity and the possibility of remote monitoring of the collected temperatures (without human intervention).

## 2. Experimental Section

The development of the sensor proposed in this work was based on the achievement of four sequential steps: proposition of the surface temperature sensor; computer simulation (for preliminary and indicative analysis of the adequacy of the proposed sensor); implementation of the proposed sensor; and validation tests. These four steps are described as follows.

### 2.1. Proposed Sensor

**Figure 1:**
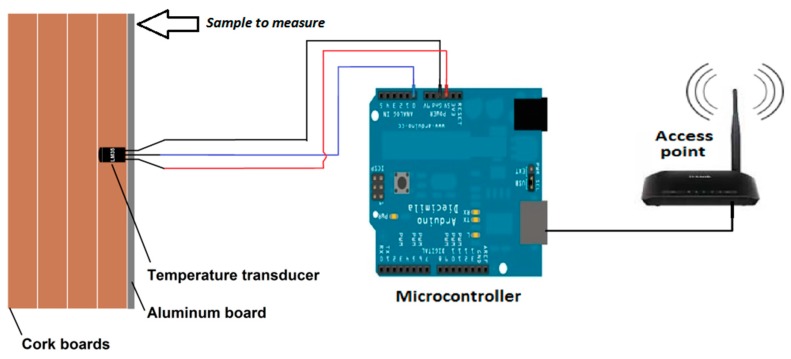
Proposed sensor.

The proposed sensor consists of four elements: a temperature transducer, a capsule (composed of aluminum and cork boards), a microcontroller, and a wireless transmission system. Figure 1 illustrates the integration of these four elements.

The temperature transducer is inserted into a capsule and is connected to a microcontroller that, after processing the measured data, sends the information to the wireless transmission system, which is responsible for transmitting the acquired information to a base or to a monitoring central.

In this work, the use of a low-cost and commercially available temperature transducer is proposed. In order of this transducer to be used as a surface temperature sensor, it must be inserted into a capsule that allows it to be fixed on different surfaces to be monitored. This capsule should be inexpensive and should enable the acquisition of surface temperature measurements without influencing the temperature of the surface being monitored; this means that this capsule should be made of a material with high thermal insulation characteristic.

The temperature transducer inserted into this capsule should then be connected to a microcontroller that is responsible for converting the information collected at the monitored surface (electrical voltage) into the measured surface temperature (°C).

After that, the measured surface temperature is sent to a wireless transmission system that can remotely transmit this information to a base or to a monitoring central. The details about each one of these four elements (temperature transducer, capsule, microcontroller, and wireless transmission system) that compose the proposed sensor are described as follows.

It is important to mention that the sensor presented in this work is proposed for indoor environments. In order to use the same sensor for outdoor environments, some adaptations must be made to make possible to expose the sensor to environmental conditions, such as heavy wind and rain.

#### 2.1.1. Temperature Transducer

The temperature transducer is responsible for “translating” electrical information (in this case, the electrical voltage measured at the monitored surface) in non-electrical information (in this case, the surface temperature). In this work, the chosen temperature transducer was the LM35, a low cost and commercially available integrated circuit (IC).

With regard to technical specifications, this IC has a TO-92 package type with three pins, namely: + Vs (input voltage), Vout (output voltage) and GND (ground). Its supply voltage can range between 4 V and 20 V and its operating range is between the temperatures of −55 °C and 150 °C [27]. The temperature transducing relation is 10 mV/°C. The main advantages of this transducer are its cost and the fact that the transducer does not require any intermediate circuit to operate.

#### 2.1.2. Capsule

As mentioned before, the LM35 is an integrated circuit with the TO-92 package type. Thus, the IC needs to be adapted to be used as a surface temperature sensor. Without this adaptation, the environment in which the transducer is collecting the surface temperature can influence the measurements. For example, the solar radiation on the structure of the IC (when it is fixed on building surfaces), or even air currents circulating in the monitored environment may interfere with the temperature measurement.

**Figure 2:**
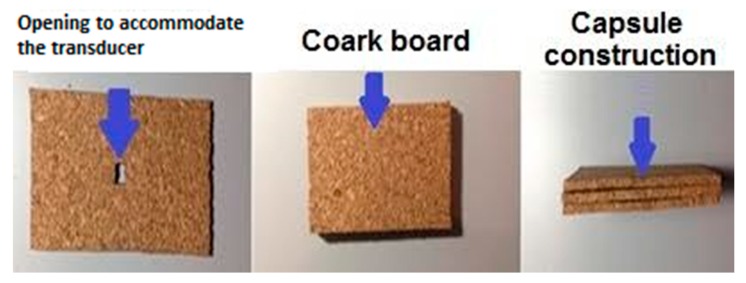
Cork boards that compose the capsule.

Hence, this work proposes the insertion of the temperature transducer into a capsule composed of four rectangular cork boards of 40 mm × 15 mm. One of these cork boards has a square opening (3 mm × 3 mm) in its center to accommodate the temperature transducer, as shown in Figure 2.

**Figure 3:**
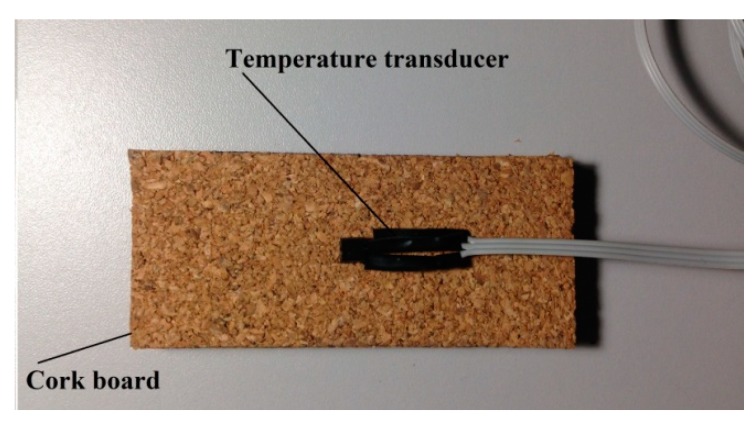
Temperature transducer positioned in the center of the fourth cork board.

Once inserted into the cork board, the temperature transducer was welded to a flat type cable with 1 m length to provide more mobility to fix the sensor on the building surface. The temperature transducer was positioned in the center of the fourth cork board, as can be seen in Figure 3.

**Figure 4:**
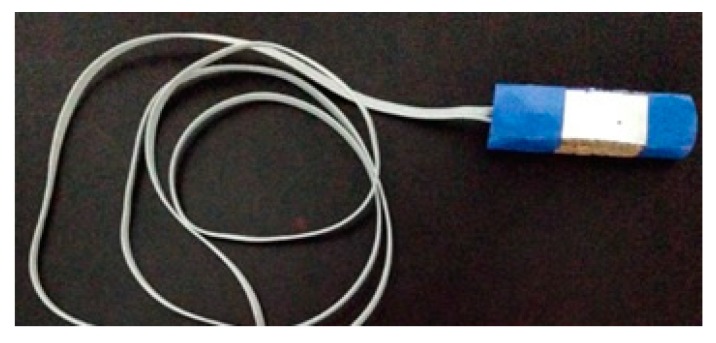
Sensor capsule.

To finish the surface temperature sensor capsule structure, an aluminum board was used due to its thermal conductivity and temperature preservation characteristics. This aluminum board has the same dimensions of the cork boards (40 mm × 15 mm), and was placed on the fourth cork board structure, smeared with a thermal paste to ensure the uniformity of the temperature in the surface under analysis. Finally, the cork boards, the aluminum board and the temperature transducer within the capsule structure were fixed with a tape of high thermal resistance. Figure 4 illustrates the sensor capsule at its final stage.

#### 2.1.3. Microcontroller

After inserted in the capsule, the temperature transducer is connected to a microcontroller responsible for performing the conversion of the voltage value measured by the transducer (Volts) in temperature values (°C). The microcontroller used in this work is the Arduino Uno, which is based on ATmega 168 processor, with six analog input pins, 1 KB of SRAM memory and an EEPROM of 512 Bytes [28].

Regarding Arduino specifications, it is important to mention that as a microcontroller, the sample frequency can be set on its firmware according to the application needs. In addition, it is possible to connect to Arduino, transceivers that, after receiving the data processed by the microcontroller, transmit them using radio frequency. In this sense, Arduino supports the connection of transceivers that operate in the most diverse frequency bands. In this work, the frequency band used was 915 MHz (IEEE 802.15.4 standard), as described in item Section 2.1.4.

With regard to other commercially available microcontrollers, the chosen model has as advantages (a) to have an affordable cost, (b) to have a wide range of sensors compatible with its operating requirements, and (c) to have free content and programming libraries in C language.

#### 2.1.4. Wireless Transmission System

After the processing of the raw data by the microcontroller, this information is sent to a wireless transmission module. The radio module (RFBee module) used for remotely transmitting the surface temperature data in wireless mode, employs the IEEE 802.15.4 standard [29].

The first version of this wireless standard (IEEE 802.15.4), was presented in 2003 (later updated in 2006), with the purpose of providing the possibility of implementing non-complex communication networks, with low cost and low energy consumption, operating in the frequency range of 915 MHz. In this standard, data management and control are performed using a random access technique, known as Carrier Sense Multiple Access with Collision Avoidance (CSMA/CA), being able to work in different network topologies such as star, point-to-point and multipoint [29].

### 2.2. Computer Simulations

Computer simulations were performed using the software ANSYS, 15.0.1.1R15.0-Academic version. These simulations were designed to investigate the influence of the mass and of the materials of the proposed sensor in the surface temperature measurements. Consequently, the objective of the simulation was to verify if the aluminum plate affects or not the temperature measurements. This analysis was carried out by observing the surface temperature behavior (as a function of time) on a sample of a common envelope technology used in buildings, when it was heated by an external source.

**Figure 5:**
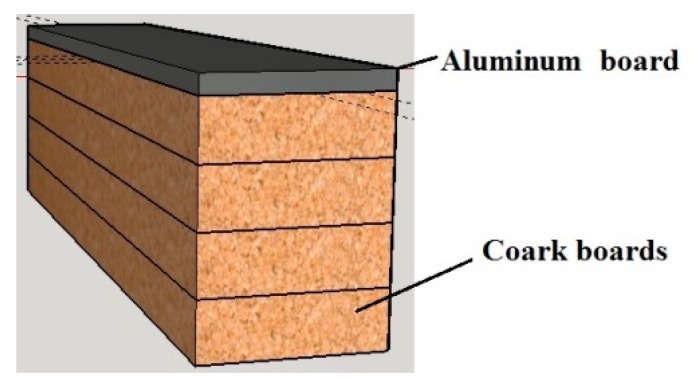
3D modeling of the capsule.

In order to reach this goal, the sensor geometry was modeled in three dimensions (3D modeling). This geometry was modeled in Auto-cad Professional 3D software, and was exported (to ANSYS) as a solid. As mentioned before, the proposed sensor consists of a temperature transducer placed in a capsule that was described in the previous section. Figure 5 illustrates, in perspective, the 3D model of the proposed capsule. In this figure, one can view the structure of the 3D modeled capsule, composed of four cork boards and an aluminum board.

**Figure 6:**
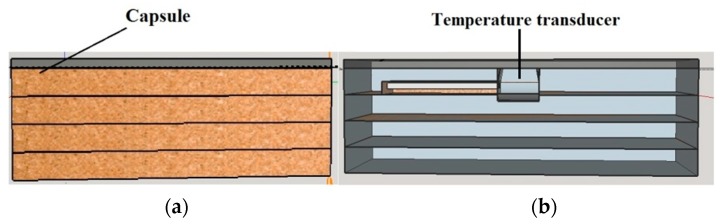
3D modeling of the proposed sensor—side view. (**a**) Proposed capsule. (**b**) Temperature transducer within the capsule.

Figure 6a illustrates a sectional side view of the proposed capsule. In Figure 6b, one can visualize the temperature transducer within the capsule.

**Figure 7:**
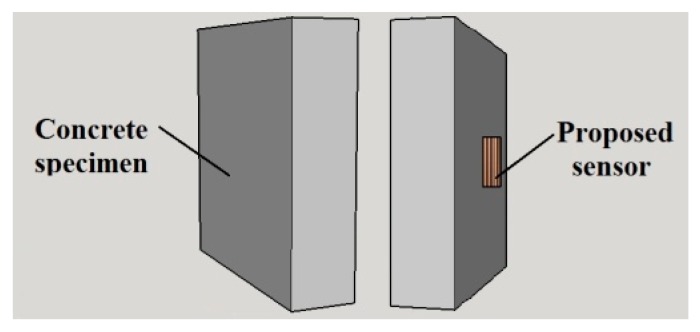
3D modeling of the concrete specimen (back surface and frontal surface, in which the sensor is placed).

The sensor proposed in this work will be used to measure the surface temperature of buildings. Consequently, in all simulations and in all validation tests, it was assumed that the proposed sensor will be used to measure the surface temperature of a concrete wall. Thus, in the simulations, a concrete specimen was modeled in 3D, with the following dimensions: 200 mm length, 200 mm high and 100 mm thick. This concrete specimen is illustrated in Figure 7. It is important to emphasize that the test bench that is used to validate the proposed sensor (Section 2.3) is composed by a concrete specimen with these same dimensions.

**Figure 8:**
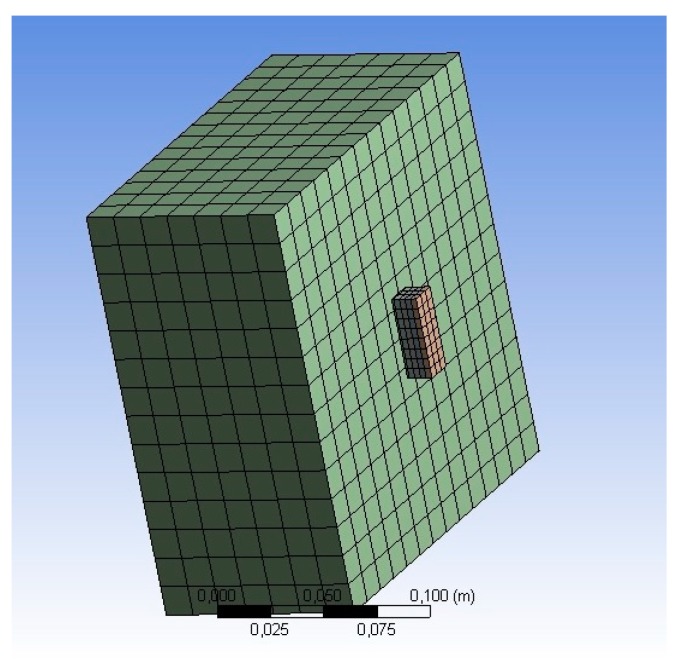
Mesh with rectangular elements that is used in the simulations.

The file with the 3D modeling of both the concrete specimen and the temperature sensor was imported into the simulation environment of the software ANSYS. This simulation software solves the conditions set in imported 3D objects, using the finite elements method (FEM) [30], which is a technique that allows the analysis of solid structures from the point of view of its strength, its flexibility, its response to heating conditions, among other applications. In order to use the FEM, it is necessary to create a mesh over the entire structure to be simulated. In Figure 8, it is possible to observe the mesh created to perform the simulations in this work.

Once the mesh was generated on the objects to be simulated, simulation parameters were set according to Table 1. It is important to emphasize that there were no other boundary conditions in the simulations.

The results of all simulations are described in Section 3.

### 2.3. Validation Tests Description

The validation of the proposed surface temperature sensor was carried out in a test bench. These validation tests were intended to verify the validity of the developed sensor based on the comparison of the obtained results (surface temperature measurements) using the proposed sensor and using a commercial temperature sensor (called reference sensor). This reference sensor corresponds to a data logger usually used for temperature monitoring, as described in references [31,32,33,34,35,36,37]. There are many models of sensors, commercially available, that are commonly used for temperature monitoring. In this work, the models of these sensors will not be mentioned to preserve the identities of the manufacturers.

**Figure 9:**
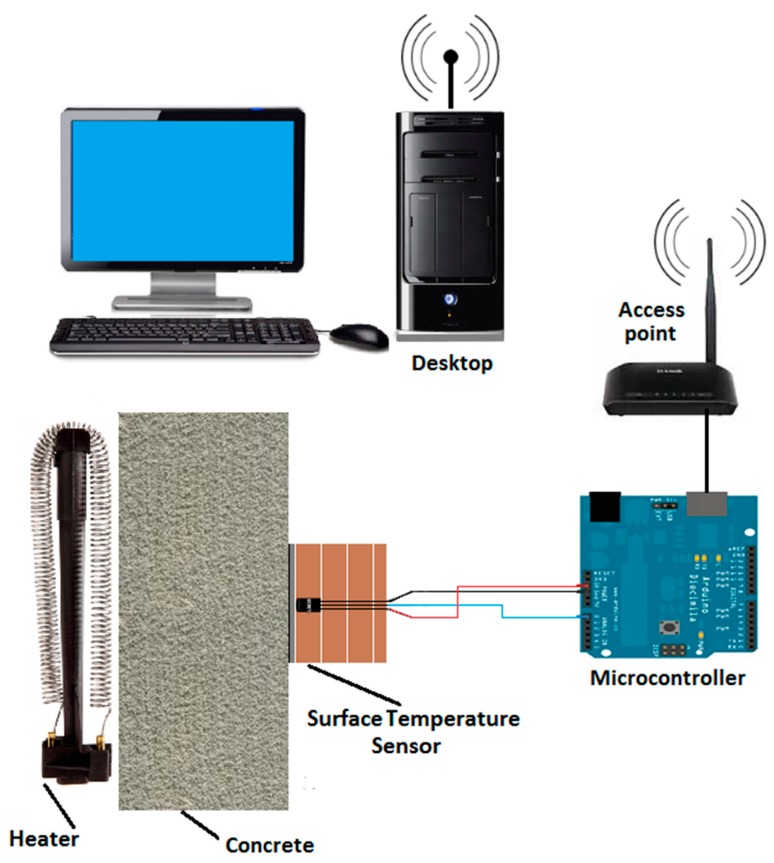
Electrical diagram of the test bench used in the validation tests.

Furthermore, in order to compare the test bench results with the computer simulations results (using the ANSYS software), the test bench implementation considered the same conditions that were set on the simulation parameters, described in Table 1. Hence, the test bench used in this work was composed by the following elements: concrete test specimen (200 mm length, 200 mm high and 100 mm thick), radios for temperature data wireless transmission, two domestic heating elements to warm the test specimen and a notebook to perform the storage of the collected data. The test bench is illustrated in Figure 9.

The test duration was 1 h with the heaters set at 90 °C (same conditions that were set in the computer simulations), and placed in a surface of the test specimen. The proposed sensor was placed in the center of the opposite surface. The surface temperature was measured at every 10 s by the proposed sensor and also by a commercial sensor (reference sensor), placed on the same surface. After 1 h, approximately, the heaters were turned off and removed from the test bench. It is important to note that to ensure that the temperature on the opposite surface reaches 90 °C, this temperature was measured using a commercially available sensor.

## 3. Results and Discussion

The results of the computer simulations and of the proposed sensor validation tests are presented in the following.

### 3.1. Computer Simulation Results

**Figure 10:**
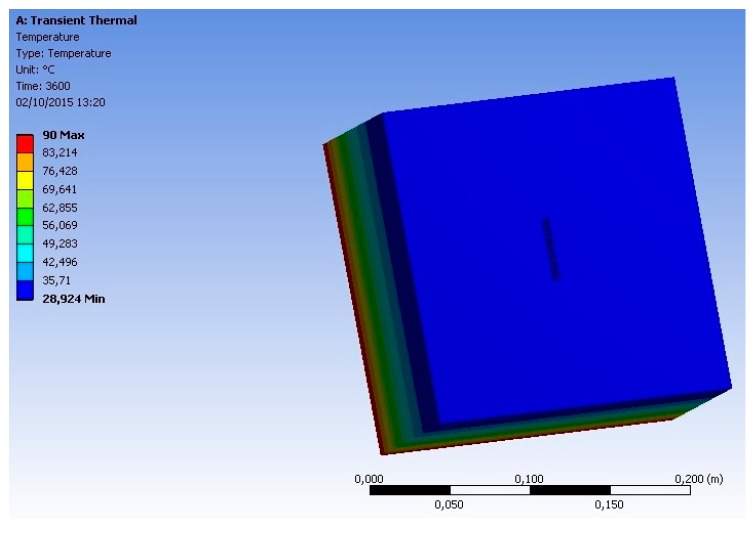
Temperature distribution through the simulated objects (concrete specimen and proposed sensor)—front view.

**Figure 11:**
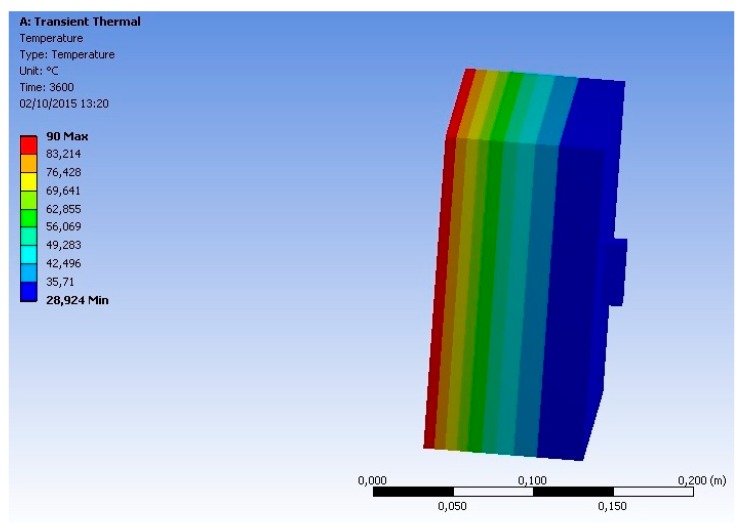
Temperature distribution through the simulated objects (concrete specimen and proposed sensor)—side view.

Computer simulations were performed according to the method described in Section 2.2. Figure 10 and Figure 11 illustrate the temperature distribution through the simulated objects (concrete specimen and proposed sensor).

From the analysis of these figures, it can be seen that the insertion of a heat source (at 90 °C) on the backside face of the geometry, implies a temperature distribution along the analyzed objects (concrete specimen and sensor). This temperature varied between 90 °C (backside face) and 28.92 °C (front face, where the sensor was positioned). It is also possible to observe that the temperature at the surface on which the sensor is positioned is uniform (the same temperature can be observed on the concrete and on the sensor). This means that the mass and the materials that compose the sensor do not influence the measurement of the surface temperature.

To verify, in a more detailed way, the influence of the sensor on the surface temperature measurements, the graphs, presented in the following figures, and based on the results of the simulations, were generated.

**Figure 12:**
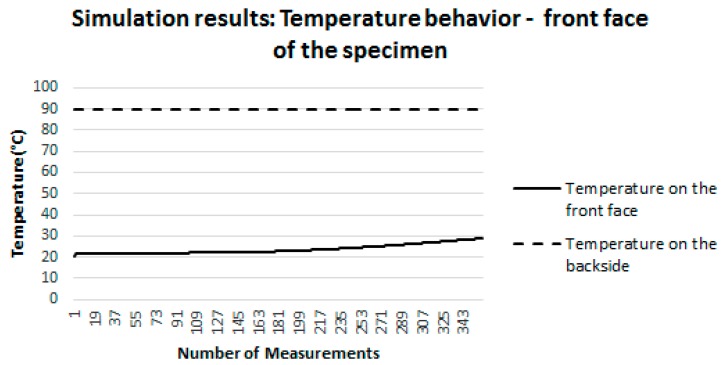
Behavior of the temperature on the front face of the specimen, in which the sensor was positioned.

Figure 12 shows the behavior of the temperature established by the heat source on the back face of the specimen (dashed curve), held constant (90 °C) throughout the simulation interval. In addition, this figure also illustrates the behavior of the temperature on the surface where the sensor is located (front face of the specimen). This behavior is represented by the continuous line. It is possible to note that the temperature on this face starts at 22 °C (ambient temperature set in the simulation) and increases until it reaches 28.92 °C.

**Figure 13:**
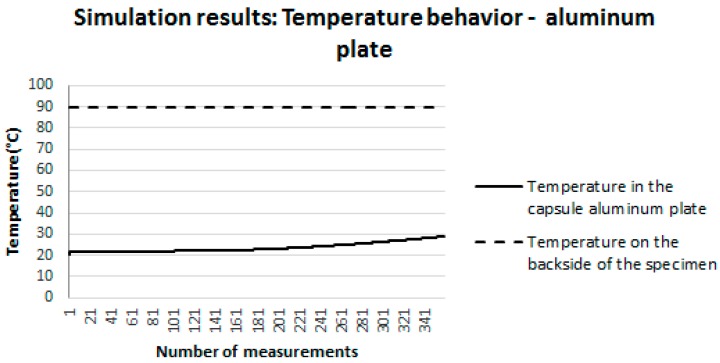
Temperature behavior in the proposed sensor’s capsule aluminum plate.

Figure 13 illustrates, one more time, the behavior of the temperature set by the heat source on the backside of the specimen (dashed curve), held constant throughout the simulation interval (90 °C). Moreover, this figure also illustrates the behavior of the temperature in the capsule aluminum plate of the proposed sensor (continuous line). It is possible to note that the temperature on the aluminum plate starts at 22 °C (room temperature set in the simulation) and increases to 28.92 °C. That is, the temperature of the aluminum plate behaved in the same way as the temperature of the surface on which the sensor was positioned (Figure 12).

It is important to mention that the difference between the temperature measured on the concrete surface and on the aluminum plate was only 1%.

**Figure 14:**
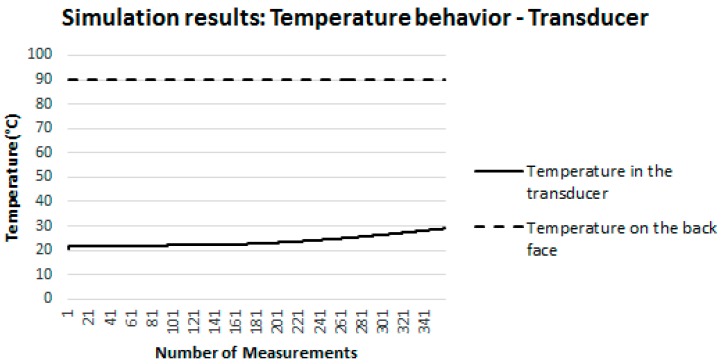
Behavior of the temperature in the transducer.

Figure 14 illustrates the behavior of the temperature established by the heat source on the back face of the specimen (dashed curve) held constant (90 °C) throughout the simulation interval. In addition, this figure also illustrates the behavior of the temperature in the transducer of the proposed sensor (continuous line). It is possible to note that the temperature in the transducer starts at 22 °C (ambient temperature established in the simulation) and increases until it reaches 28.92 °C. That is, the temperature of the transducer behaved in the same way as the temperature of the surface on which the sensor was positioned (Figure 12).

It is important to emphasize that the difference between the temperature measured on the concrete surface and on the transducer was only 2%. From the analysis of Figure 12, Figure 13 and Figure 14, it can be concluded that, in the simulations carried out, the mass and the materials component that compose the sensor do not influence the surface temperature measurement. This conclusion allows attesting the suitability of the proposed sensor for the measurement of surface temperatures in buildings.

### 3.2. Validation Tests Results

**Figure 15:**
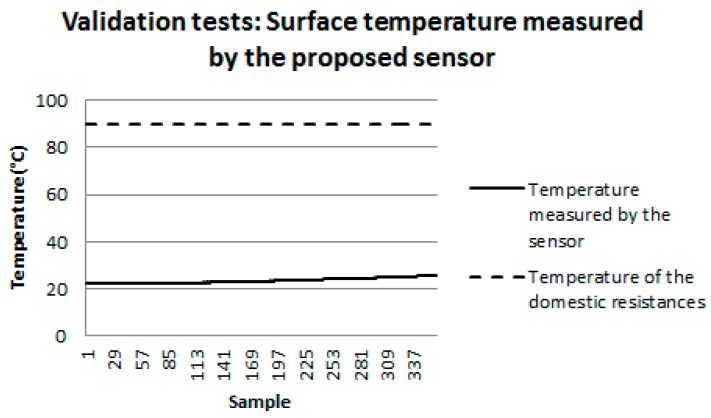
Surface temperature measured by the proposed sensor.

As previously mentioned, the validation tests were performed using a bench, developed according to the method already described in Section 2.3. Figure 15 shows the behavior of the temperature of the domestic resistances used to heat the back face of the test specimen (dashed curve). It can be noted that this temperature remained constant (90 °C) throughout the test period. The continuous line represents the behavior of the surface temperature at which the sensor was positioned, and this temperature was measured by the sensor proposed in this work.

It is possible to observe that the surface temperature measured by the proposed sensor starts at around 22 °C and increases until reaching the value of 25.41 °C. Comparing these results with the results obtained in the computational simulations (Figure 12), it was verified that the percentage error between the final temperature on this surface measured by the proposed sensor (25.41 °C) and computationally simulated (28.92 °C) was 13%. This percentage error can be explained by the fact that, unlike what happens in the computational simulation, the environment in which the test bench was inserted was not controlled, and there were changes in the environment such as entry and exit of people and opening and closure of doors and windows. However, despite differences in measured and simulated temperatures, it can be seen that the behavior of the surface temperature over time is similar, that is, it increases over time.

**Figure 16:**
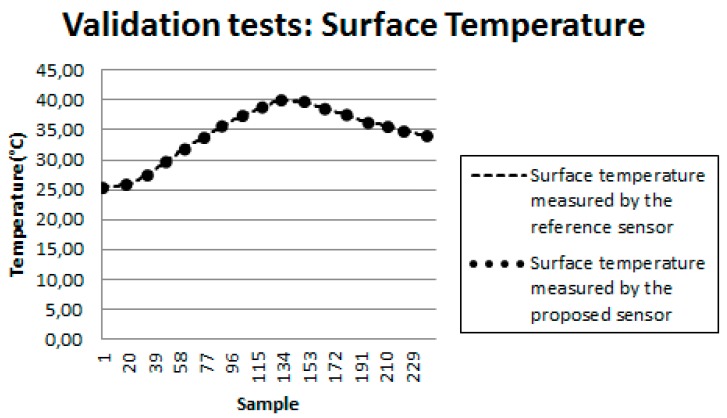
Surface temperature.

**Figure 17:**
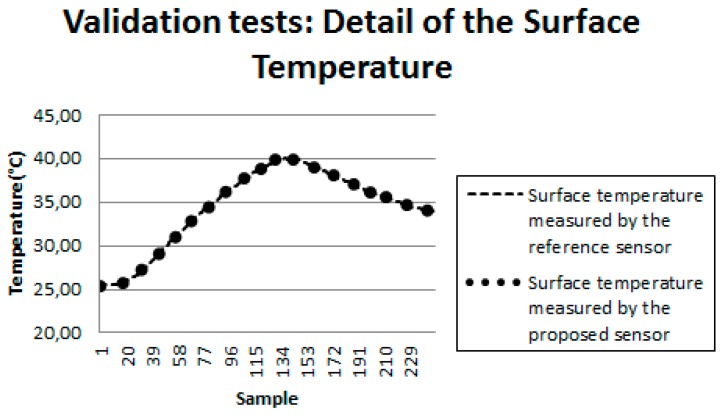
Detail of the surface temperature.

Figure 16 shows the results obtained for another test with the developed bench. In this figure, the curve represented by circles illustrates the behavior of the surface temperature measured by the proposed sensor, while the dashed curve illustrates the same temperature measured by the reference sensor (conventional commercial sensor). Figure 17 presents a detail of Figure 16.

After analyzing the collected data, the mean percentage error between the measurements made by the proposed sensor and the reference sensor was calculated according to Equation (1):

(1) $$MPE=\frac{1}{N}\cdot \sum_{i=1}^{N}\frac{\left|Tsp_{i}-Tref_{i}\right|}{Tref_{i}}$$

where *MPE* is the Mean Percentage Error; *N* is the total number of measurements; *i* corresponds to measurement “*i*”; *Tsp* is the temperature measured by the proposed sensor; *Tref* is the temperature measured by the reference sensor. In the tests performed, the calculated *MPE* was 2%, attesting the validity of the sensor developed in this work.

### 3.3. Cost Analysis

A cost analysis was also performed, comparing the proposed sensor and a sensor commercially available and conventionally used for surface temperature monitoring (the reference sensor was described in Section 2.3).

As cited before, in this work, the models of the sensors conventionally used for temperature monitoring, will not be mentioned to preserve the identities of the manufacturers. Table 2 illustrates the cost of two sensor models (sensor A and sensor B), that are conventionally used for temperature measurement, and of the proposed sensor.

From a broader survey conducted with manufacturers of this type of sensor, it was possible to determine an average cost related to this device. On average, the reference sensor can be acquired by, approximately, US$ 234.00 and the cost associated to the proposed sensor is, approximately, US$ 91.00, which represents a cost saving of approximately 61% compared to the commercial sensor.

The prototype developed in this work has the possibility of expanding to up to eight sensors. This means that it is possible to use 8 transducers (inside cork capsules) and only one microcontroller and one wireless transmission system. Thus, considering the possibility of using eight surface temperature sensors in a building, the cost associated to these eight sensors would be US$ 144.00. Considering the use of eight reference sensors (the reference sensor was described in Section 2.3), that do not have expansion possibilities, the associated cost would be US$ 1872.00 (8 × US$ 234.00), which corresponds to eight times the average cost of this sensor. That is, the use of eight sensors proposed in this work represents a cost saving of approximately 92% with respect to the use of eight reference sensors. Thus, it can be considered that the developed sensor was able to adjust to the low cost requirement, set initially as a key feature in this development.

### 3.4. Results of the Wireless Transmission System

The data presented in Section 3 were measured by the transducer proposed in this paper and transmitted using the IEEE 802.15.4 standard. The proposed wireless transmission system was able to transmit the collected data without loss of information or significant degradation of the signal.

In the tests carried out to validate the adopted wireless transmission system, some parameters related to the Quality of Service of the proposed communication network were measured, such as attenuation and throughput. Table 3 shows these measured parameters.

These measured parameters show that the adopted wireless transmission system allows the remote monitoring of the surface temperature in buildings, since they indicate that de transmitted signal does not suffer quality degradation.

## 4. Conclusions

This work proposed and presented the implementation of a surface temperature sensor for use in the built environment. The sensor met the following requirements: low cost, large data storage capacity (since the collected temperatures are stored in a monitoring station/notebook) and possibility of remote monitoring of collected data (using a wireless transmission system).

Initially, computational simulations were performed to verify the influence of the mass and the material of the proposed sensor on the surface temperature measurements. In this sense, the results obtained from these simulations demonstrated that the mass and the material of the proposed sensor do not influence the measurements made.

Since the computational simulations proved the suitability of the proposed sensor, it was implemented and tested using a test bench, aiming its validation. In this bench, the measurements of surface temperature using the proposed sensor were compared with the measurements made using a reference sensor (commercially available). The results obtained from the tests carried out on this bench showed the validity of the proposed sensor, since the mean percentage error was 2%, considering the commercial sensor as reference.

In addition, a cost analysis was performed in which the advantage of using the proposed sensor is evident. This analysis demonstrates that the use of eight surface temperature sensors (as proposed in this work) in a building implies a cost savings of approximately 92% in relation to the use of eight commercial sensors.

Moreover, the proposed sensor provides the wireless transmission of collected data, allowing the remote monitoring of buildings. This monitoring can enhance energy efficiency actions in the built environment and in the urban environment, and may, for example, assist in the adequacy of buildings to requirements established in the energy efficiency rating systems, such as AQUA, LEED and PROCEL Edifica.

## Figures and Tables

**Table 1:** Simulation parameters.

Parameter	Set Value
Room temperature	22 °C
Period of analysis	1 h (3600 s)
Surface temperature on the opposite side of the sample	90 °C

**Table 2:** Cost comparison.

	Sensor A	Sensor B	Proposed Sensor
**Cost (US$)**	170.00	240.00	91.00

**Table 3:** Mean values of attenuation and throughput of the wireless transmission system.

Attenuation (dBm)	Throughput (MB/s)
−49.5	30
